# Thoracolumbar spine injury in Cameroon: etiology, management, and outcome

**DOI:** 10.1186/s12891-023-06481-z

**Published:** 2023-05-15

**Authors:** Paul Chinonso Shu, Mathieu Motah, Daniel Gams Massi, Yannick Lechedem Ngunyi, Ngenge Michael Budzi, Alain Chichom Mefire

**Affiliations:** 1 Faculty of Health Sciences, University of Buea, Buea, Cameroon; 2 Faculty of Medicine and Pharmaceutical Sciences, University of Douala, Douala, Cameroon; 3 Neurology unit, Douala General Hospital, Douala, Cameroon; 4 Mbonge District Hospital, Mbonge, Cameroon; 5 Cameroon Baptist Convention health services, Yaoundé, Cameroon; 6 Douala Gyneco-obstetric and pediatric hospital, Douala, Cameroon

**Keywords:** Thoracolumbar spine injury, Road traffic accident, Management, Prognosis, Cameroon

## Abstract

**Background:**

Thoracolumbar spine injury (TLSI) is a major concern worldwide despite its low prevalence. Studies demonstrate a gradual rise in annual incidence. There have been improvements in its management. However, a lot is still to be done. TLSI secondary to trauma usually occurs abruptly and leaves demeaning consequences, especially in our setting where the prognosis from several studies is poor. This study aimed to describe the etiology, management principles, and prognosis of TLSI in Douala General Hospital and as such contribute data on those aspects in the research community.

**Method:**

This was a hospital-based five-year retrospective study. The study population was patients treated for TLSI in the Douala General Hospital from January 2014 to December 2018. Patients’ medical records were used to retrieve data. Data analysis was done using SPSS Version 23. Logistic regression models were fitted to assess the association between dependent and independent variables. Statistical significance was set at 95% CI, with a P-value < 0.05.

**Results:**

We studied a total of 70 patients’ files including 56 males. The mean age of occurrence of TLSI was 37.59 ± 14.07 years. The most common etiology was road traffic accidents (45.7%) and falls (30.0%). Half of our patients (n = 35) had an incomplete neurological deficit (Frankel B – D). Paraplegia was the most common motor deficit (42.9%). The lumbar spine was affected in 55.7% of cases. The most common CT scan finding was fracture of the vertebrae (30%) while the most reported MRI finding was disc herniation with contusion (38.5%). More than half (51.4%) of our patients were referred from peripheral health centers. The median arrival time was 48 h (IQR: 18–144) with 22.9% reporting after a week post-injury. Less than half (48.1%) benefited from surgery, and 41.4% of our population benefited from in-hospital rehabilitation. The median in-hospital delay time for surgery was 120 h (IQR: 66–192). While the median time between injury and surgery was 188 h (IQR: 144–347). The mortality rate was 5.7% (n = 4). Almost all (86.9%) of the patients developed complications and we had a 61.4% improvement in neurological status upon discharge. Being covered by health insurance was a predictor of improved neurological status (AOR = 15.04, 95%CI:2.90–78.20, P = 0.001) while being referred was a predictor of a stationary neurological status upon discharge (AOR = 0.12, 95%CI:0.03–0.52, P = 0.005). The average hospital stay was 20 days. We did not identify any predictors of lengthy hospital stay.

**Conclusion:**

Road traffic accident is the most common etiology of TLSI. The arrival time to a neurosurgery specialized center after a traumatic injury, and the in-hospital delay time for surgery is high. Reduction of these delays, encouraging universal health insurance coverage, and improving on management to reduce complications would better the outcome of TLSI which is comparable with those in other studies.

## Background

Thoracolumbar Spine Injury (TLSI) represents damage to the thoracic and/or lumbar region of the spine caused by trauma or an external force [1]. Traumatic causes include road traffic accidents (RTA), falls, and violence [2–4]. Unlike non-traumatic causes, the traumatic causes of spine injury are more devastating because they are abrupt, occur without warning, and affect mostly the young [1, 3]. Thus, the consequences are significant for the patient (due to a lifestyle change), their families, and the economy as a whole. The thoracolumbar segment is the most common site of trauma to the spine [5]. A Nigerian study reported a rising incidence of this condition, from 16 to 59 cases (1992 to 2006) [5]. The management protocols are often conservative and expensive, and many people cannot afford them [1]. The prognosis of Traumatic Spine Injury is still very poor in low-income countries as compared to high-income countries partly due to the lack of adequate facilities in place to manage this condition [1, 5]. High rates of poverty in low-income settings make it difficult to seek expensive and prolonged management options in areas where some form of adequate management is available [1]. It, therefore, reveals the need for primary and secondary prevention. Mortality rates are still high in developing countries. Another study in Nigeria revealed a mortality rate of about 17.5% [5]. Thoracolumbar spine trauma has better survival rates [6]. This is because it rarely impinges on respiration which occurs in cervical spine trauma and is one of the striking causes of death in patients with traumatic spinal cord injury [5]. Since most patients with TLSI survive, improving survival prognosis and quality of life post-trauma is imperative. This is also challenging, due to the scarcity of data available on TLSI in our setting. This study aimed to determine the etiologies, management, and prognosis of this severe condition, and to provide baseline data for directing efforts toward prevention and improving the prognosis of TLSI.

## Methods

### Study design and setting

This was a hospital-based retrospective study carried out at the Douala General Hospital. Douala is in the Wouri Division of the Littoral Region, and it is the economic capital of Cameroon. It has a seaport which is the main port in Cameroon for business. Many people converge in the city daily for business purposes, making the influx and outflux via road very high. The high rate of rural exodus to this city explains the high rate of urban mess and criminality [7]. RTA and violence are frequent etiologies of TLSI [8]. The Douala General Hospital is a tertiary hospital in the Cameroon health care system with an inherent capacity to manage conditions that cannot be managed at the periphery. It has 320 beds with a fully functional imaging and surgical unit. The hospital comprises specialized staff for the management and follow-up of TLSI patients.

### Study population and sampling

We included medical files of all patients treated in the surgical ward of the Douala General Hospital for TLSI, between 1st January 2014, and December 31st, 2018. Patients with associated cervical spine injuries or any other major injured system, and incomplete files were excluded.

### Data collection

Data were collected using a predesigned data collection sheet from patients’ medical records at the emergency, surgical, theatre, and outpatient department units. Data collection sheets were checked each time for completeness, and the use of correct coding. The data collection sheets were stored in a safe place before analysis. Names were not used as the data collection sheets were coded.

### Definition of operational terms

We defined conservative treatment as, any nonsurgical treatment. Improvement was defined as any unit change in grade. In-hospital mortality, neurological status upon discharge, lengthy hospital stay (hospital stay days greater than 30 days) and in-hospital complications with neurogenic pain (defined as pain out of proportion in the context of suspected spine injury) were our outcome variables.

### Statistical analysis

Data collected were coded, entered into Epi-info version 7.2, and exported into SPSS version 23 for analysis. Categorical variables were presented as frequencies and their proportions. Continuous variables were presented as means (and standard deviation) or median (and inter-quartile range), or as frequencies and percentages after categorizing using predefined cutoffs or the median. Bivariate logistic regression analysis was used to test the association between the dependent variable and independent variables. Statistical significance was set at a 95% confidence interval and a p-value less than 0.05.

### Ethics considerations

The ethical clearance for this study was issued by the Institutional Review Board of the Faculty of Health Sciences, University of Buea (ref. N^o^: 2019/934-01/UB/SG/IRB/FHS). Administrative approval was obtained from the Director of Human Resources of the Douala General Hospital. To ensure confidentiality, all patient information was coded.

## Results

We reviewed 222 patients with Traumatic Spinal Cord Injury including 110 (49.55%) cases of TLSI with increased incidence over the years: 2014 (n = 6), 2015 (n = 14), 2016 (n = 18), 2017 (n = 14), and 2018 (n = 18). Seventy (70) files fulfilled the inclusion criteria and were selected for this study.

**Table 1:** Socio-demographic characteristics and mechanisms of TLSI

Variables		n	%
Age group (years)			
	< 21	3	4.3
	21–40	42	60.0
	41–60	21	30.0
	> 61	4	5.7
Gender			
	Female	14	20.0
	Male	56	80.0
Job Description			
	Skilled	33	47.1
	Unskilled	21	30.0
	Unemployed/Student	16	22.9
Mode of payment			
	Out of pocket	36	51.4
	Health insurance	34	48.6
Mechanisms of trauma			
	Road Traffic Accident	32	45.7
	Falls from a height	21	30.0
	Work-related	10	14.3
	Violence	4	5.7
	Home related	2	2.9
	Sports-related	1	1.4

### Socio-demographic characteristics and mechanisms of TLSI

The mean age was 37.59 ± 14.07 years with male predominance (80%, n = 56). The majority (77.1%, n = 51) were employed in unskilled jobs including farming, driving, and retailing. RTA (45.7%, n = 32) and falls (30%, n = 21) were the most common mechanisms of TLSI (Table 1).

### Clinical and neuroimaging features

Frankel E (30%, n = 21) was the commonest clinical presentation followed by Frankel A (20.0%, n = 14). Half (50%, n = 35) of the patients had incomplete spinal cord syndrome. The most common motor deficit was paraplegia (42.9%, n = 30). A spine CT scan was done in all cases, and 13 patients did a spine MRI. Fracture of the vertebrae (arch, body, or both) was the commonest CT scan finding (32.9%). The lumbar spine (55.7%, n = 39) was the most affected region while 7 (10%) of patients had thoracic and lumbar spine involvement(T10-L2) hence multisegment injury. (Table 2).

**Table 2:** Clinical and neuroimaging features

Variables		n	%
Frankel’s			
	A	14	20.0
	B	13	18.6
	C	13	18.6
	D	9	12.8
	E	21	30.0
Motor deficit			
	Paraparesis	15	21.4
	Paraplegia	30	42.9
	Monoplegia	4	5.7
	None	21	30.0
Spinal regions			
	Thoracic	24	34.3
	Lumbar	39	55.7
	Thoracolumbar	7	10.0
Spine CT scan (n = 70)			
	Fracture	23	32.9
	Fracture + compression	21	30.0
	Normal	14	20.0
	Unknown	12	17.1
Spine MRI (n = 13)			
	Contusion	8	61.5
	Compression	4	30.8
	Disc herniation	5	38.5
	Normal	1	7.7

CT: Computed tomography, MRI: Magnetic resonance imaging

### Pre-hospital and in-hospital management of patients with TLSI

**Fig. 1:**
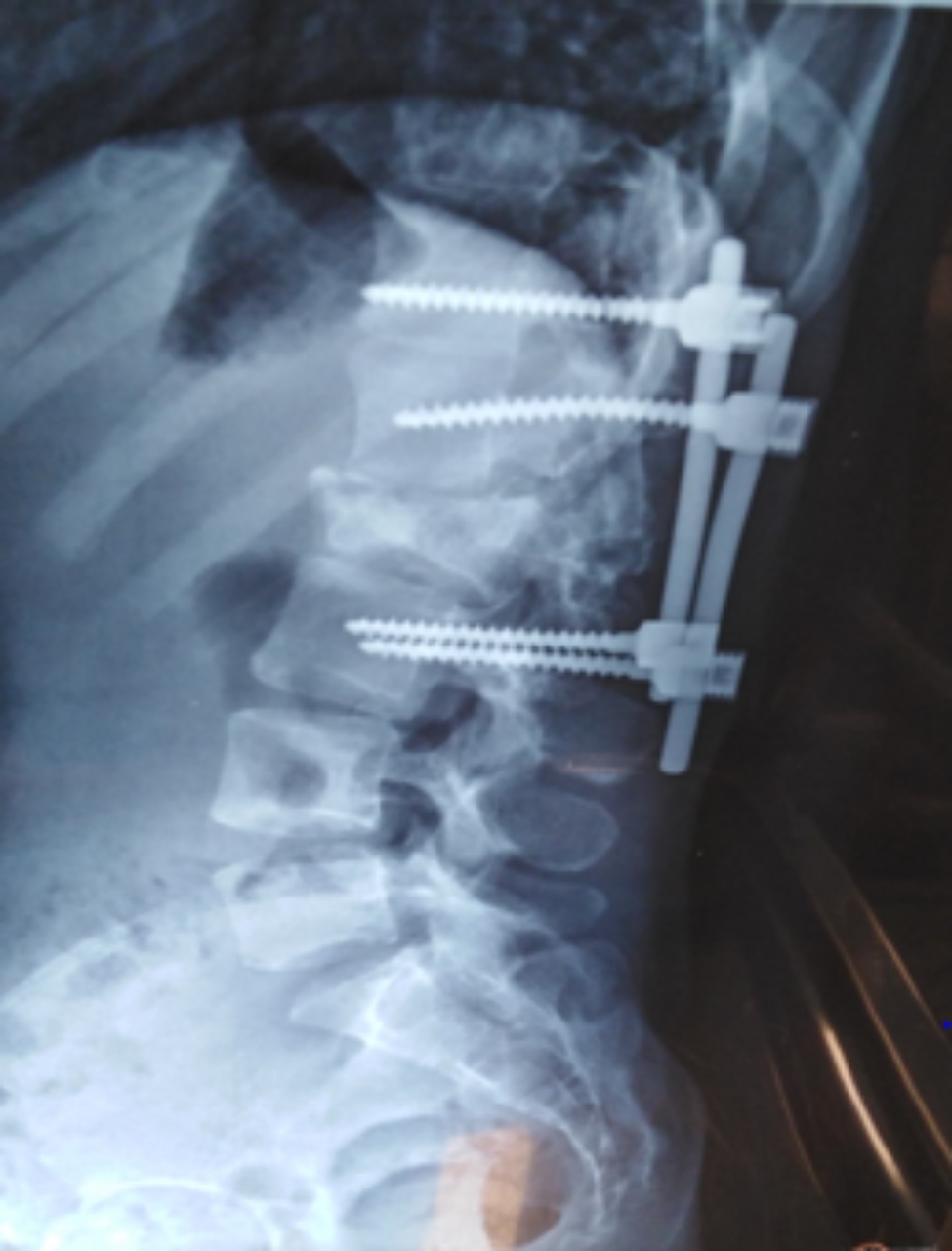
Fixation of L3-L1-T12 for a fracture of L2 (lateral view)

Transportation to DGH was done through a non-ambulance vehicle in most cases (90%, n = 63). Most of the patients (51.4%, n = 36) were referred from a primary care center. Three treatment options were available: medical (100%, n = 70), orthopedic (10%, n = 7), and surgical (48.6%, n = 34) (Table 3). The main surgical procedure was laminectomy + fixation (67.6%, n = 23) (Fig. 1).

All fixations were done through the posterior approach using the Coterl-Dubosset and Roy-Camille materials. All the patients received steroids, and analgesics (Diclofenac and Tramadol). The median time between injury and arrival was 48 h (IQR: 18–144) with a mean arrival time of 400 h. Sixteen (22.9%) patients arrived in the hospital more than 1 week after injury (Table 3).

**Fig. 2:**
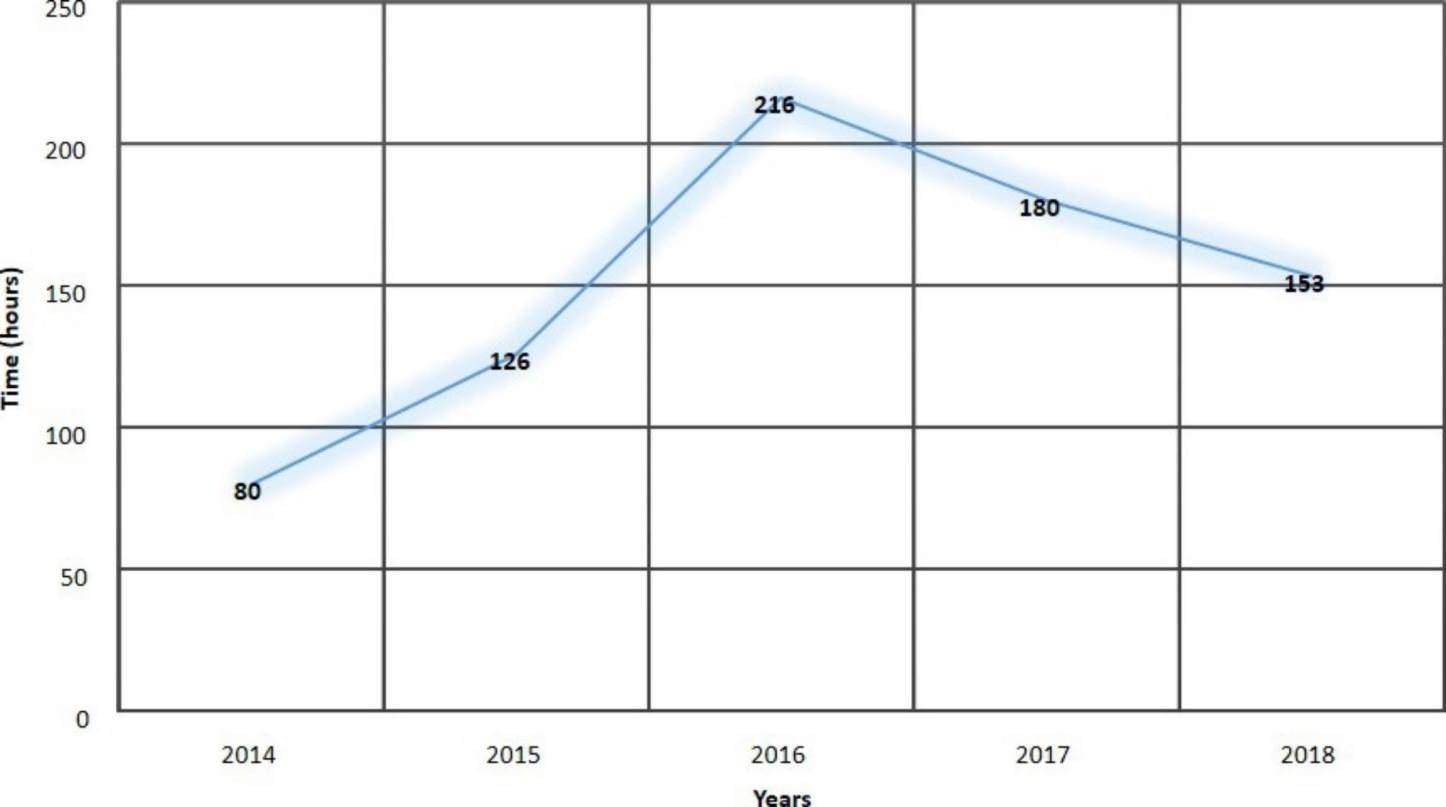
Trends in in-hospital delay time for surgery over the five years of study

The median time from arrival to surgery in our institution was 120 h (66–192) with most patients (55.9%, n = 19) benefiting from surgery between the second and the seventh days of admission. The median time between injury and surgery was 188 h (144–347). Most patients (55.9%, n = 19) had surgery more than a week after injury (Table 3). The timing between arrival and surgery in this institution has fluctuated over the years with a decline in the last two years (Fig. 2).

**Table 3:** Pre-hospital and In-hospital management of patients with TLSI

Variables		N	%
Arrival time in days(hours)			
	≤ 1(24)	32	45.7
	> 1 to 7(24–168)	22	31.4
	> 7(> 168)	16	22.9
Means of Transport			
	Non-ambulance	63	90.0
	Ambulance	7	10.0
Referred			
	Yes	36	51.4
	No	34	48.6
Medical			
	Analgesics	70	100.0
	Corticosteroids	70	100.0
	LMWH	60	85.7
	Physiotherapy	29	41.4
	ICU management	3	4.3
Orthopedic	Lumbar corset	7	10.0
Surgical		34	48.6
	Laminectomy	11	15.7
	Laminectomy + fixation	23	32.9
Time: ED to surgery in days(hours)			
	< 1(< 24)	5	14.7
	1–7(24–168)	19	55.9
	> 7(> 168)	10	29.4
Time: injury to surgery in days(hours)			
	< 2(< 48)	0	0.0
	2–7(48–168)	15	44.1
	> 7(> 168)	19	55.9

ED: Emergency department

### In-hospital complications of patients with TLSI

The in-hospital mortality following TLSI was 5.7% (n = 4), with the most common cause of death being respiratory failure. A total of 58 (86.9%) patients developed complications during their hospital stay. The most common complication was: Neurogenic pain (77.1%, n = 54) followed by depression (44.3%, n = 31) (Table 4).

**Table 4:** In-hospital complications of patients with TLSI.

Characteristic	Frequency (n = 70)	Percentage (%)
**In-hospital Death**	4	5.7
**Cause of Death**		
Respiratory failure	2	50.0
CNS	1	25.0
CVS	1	25.0
**Complications**		
Neurogenic Pain	54	77.1
Depression	31	44.3
Constipation	11	15.7
Wound infection	8	11.4
Bedsores	7	10.0
UTI	3	4.3

CNS: Central nervous system, CVS: Cardiovascular system, UTI: Urinary tract infection

### Neurological status upon discharge of patients with TLSI

We had a 61.4% (n = 43) improvement rate, out of which 76.7% (n = 33) were complete recovery. (Table 5). The median hospital stay was 11 days (IQR:5–26.5), the average being 20 days. Most patients (47.1%, n = 33) went home before 10 days.

### Comparison of types of complications in the various neurological deficit

There was an association between the severity of injury and the type of complications we could expect. Bedsore was significantly associated with complete injury as 71.4% of those who had bedsore had a complete injury (p = 0.001). Patients with complete injury were also more likely to be depressed or need psychological support/therapy. (Table 5)

**Table 5:** Comparison of types of complications in the various neurological deficit **(Bivariate analysis)**

Complication	Neurological Deficit			P value
	Complete	Incomplete	Normal	
**Neurogenic Pain**				0.806
Yes	10	28	16	
No	4	7	5	
**Wound infection**				0.139
Yes	2	6	0	
No	12	29	21	
**Bedsore**				**0.001**
Yes	5	2	0	
No	9	33	21	
**UTI**				0.498
Yes	1	2	0	
No	13	33	21	
**Depression**				**< 0.001**
Yes	12	18	1	
No	2	17	20	
**Constipation**				0.061
Yes	3	8	0	
No	11	27	21	

UTI: Urinary tract infection

### Association between management principles and neurological status upon discharge

There was no association between management and mortality. However, all three patients admitted to the ICU died. Those who first went to another institution before presenting at our institution were less likely to have an improved neurological status upon discharge than those who came directly (78.1% vs. 52.9%, OR (95%CI): 0.32(0.11–0.92), p = 0.032). (Table 6)

**Table 6:** Association between management principles and neurological status upon discharge (Bivariate analysis)

Management Principle	Neurological status upon discharge		OR (95%)	P-Value
**N = 66**	**Stationary**	**Improved**		
**Means of transport**				
Ambulance	1(14.3)	6(85.7)	3.57 (0.40–3.62)	0.227
Non-ambulance	22(37.3)	37(62.7)	1	
**Referred**				
Yes	16(47.1)	18(52.9)	0.32 (0.11–0.92)	**0.032**
No	7(21.9)	25(78.1)	1	
**Mode of Payment**				
Out of pocket	19(55.9)	15(44.1)	1	
Insurance	4(12.5)	28(87.5)	8.87(2.55- 30.87)	**< 0.001**
**Surgery**				
No	13(40.6)	19(59.4)	0.61(0.22- 1.69)	0.339
Yes	10(29.4)	24(70.6)	1	
**Injury to Surgery**				
≤ 1 week	7(53.8)	6(46.2)	1	
>1 week	6(31.6)	13(68.4)	2.53(0.59- 10.86)	0.208
**Psychotherapy/Counselling**				
Yes	13(30.2)	30(69.8)	1.78(0.62- 5.07)	0.282
No	10(43.5)	13(56.5)	1	
**In-hospital** **Rehabilitation**				
Yes	6(20.7)	23(79.3)	3.26(1.08- 9.86)	**0.033**
No	17(45.9)	20(54.1)	1	

Significant level: P < 0.05

### Management predictors of neurological status upon discharge

Following multivariate analysis with variables that were significantly associated with neurological status upon discharge, referral was a predictor of a stationary neurological status upon discharge while paying through insurance was predictive of an improved status upon discharge. (Table 7)

**Table 7:** Management predictors of neurological status upon discharge (Multivariate Analysis)

Variable	P-value		Adjusted OR (95% CI)
Referred	**0.005**		0.12(0.03–0.52)
In-hospital Rehabilitation	0.426		1.76(0.44–7.06)
Insurance	**0.001**		15.04(2.90–78.20)

Significant level: p < 0.05

### Association between management and lengthy hospital stay

Patients referred were more likely to stay longer in the hospital than those who came directly (30.6% vs. 8.8%, p = 0.023). Having surgery, injury to surgery time greater than a week, and having in-hospital rehabilitation were also associated with lengthy hospital stays. (Table 8).

**Table 8:** Association between management and lengthy hospital stay (Bivariate analysis)

Management Principle	Hospital Stay > 30 days		OR (95% CI)	P- value
**N = 70**	NO	YES		
**Means of transport**				
Ambulance	7(100.0)	0(0.0)	NA	0.103
Non-ambulance **Referred**	49(77.8)	14(22.2)		
Yes	25(69.4)	11(30.6)	4.55(1.14–18.09)	**0.023**
No **Mode of Payment**	31(91.2)	3(8.8)	1	
Out of pocket	30(83.3)	6(16.7)	1	
Insurance **Surgery**	26(76.5)	8(23.5)	1.54(0.47–502)	0.473
Yes	21(61.8)	13(38.2)	21.67(2.64177.76)	**< 0.001**
No	35(97.2)	1(2.8)	1	
**Injury to Surgery**				
≤ 1 week	13(86.7)	2(13.3)	1	
>1 week **Psychotherapy**	8(42.1)	11(57.9)	8.94(1.56–51.18)	**0.008**
Yes	33(73.3)	12(26.7)	4.18(0.85–20.48)	0.061
No **In-hospital** **Rehabilitation**	13(86.7)	2(13.3)	1	
Yes	18(62.1)	11(37.9)	7.74(1.92–31.21)	**0.002**
No	38(92.7)	3(7.3)	1	

Significant level: P < 0.05, N/A: Non-Applicable

### Association between complications and lengthy hospital stay

All complications except UTI were significantly associated with a lengthy hospital stay. (Table 9). Following multivariate analysis, we found no predictors of hospital stay.

**Table 9:** Association between complications and lengthy hospital stay (Bivariate analysis)

Complication	Hospital stay > 30 days		OR (95%CI)	P value
**N = 70**	NO	YES		
**Neurogenic Pain**				
Yes	40(74.1)	14(25.9)	N/A	**0.023**
No **Wound infection**	16(100.0)	0(0.0)		
Yes	3(37.5)	5(62.5)	9.82(1.99–48.43)	**0.001**
No **Bedsore**	53(85.5)	9(14.5)	1	
Yes	3(42.9)	4(57.1)	7.05(1.37–36.52)	**0.010**
No **UTI**	53(84.1)	10(15.9)	1	
Yes	2(66.7)	1(33.3)	2.08(0.18–24.69)	0.555
No **Depression**	54(80.6)	13(19.4)	1	
Yes	21(67.7)	10(32.3)	4.17(1.16–14.98)	**0.022**
No **Constipation**	35(89.7)	4(10.3)	1	
Yes	6(54.5)	5(45.5)	4.63(1.16–18.45)	**0.022**
No	50(84.7)	9(15.3)	1	

Significant level: P < 0.05, N/A: Non-Applicable

## Discussion

In this study, TLSI affected mainly males of young age. This was consistent with several studies which found a high occurrence in males than in females [9–11]. It affects mostly the younger population probably because they are most active in society. Therefore, it has a burden on the economy as it affects the productive population. In Cameroon, jobs highly exposed to trauma are led by young men, such as motorcycle-taxi. RTA and falls represented the most frequent mechanisms of TLSI. These findings are consistent with many studies in Africa [5, 12–14]. This could be explained by the bad state of our roads which Sobngwi et al. in 2010 found to be one of the causes of RTA [15]. In addition, very few safety measures are respected by motorcycle-taxi users.

One patient out of five presented a complete neurological deficit (Frankel A). This finding is consistent with the findings of Reinhold et al. (Germany, 2010) where the majority of patients (74%) had incomplete neurological deficits [16]. However, a high frequency of Frankel A cases (91%) was reported by Obalum et al. (Nigeria, 2009) [5]. This is probably because their study also included patients with cervical spine injuries. The injury was located in the lumbar spine in more than half of the cases. This was consistent with the findings of Obalum et al. (Nigeria, 2009) which found lumbar trauma in about 60% of cases [5]. The lumbar spine is easily affected more than the thoracic spine because the thoracic spine has the rib cage which gives it more stability. In this study, one patient out of ten had multi-segment spine trauma. Reinhold et al. (Germany, 2010) reported multi-segment involvement in 8% of patients [16].

The timing of arrival at the hospital is an important feature in pre-hospital management [1]. It is recommended for such patients to get to the hospital within 24 h after injury [17]. The mean arrival time of patients after injury in this study was 400 h (16 days). More than half of our patients came more than one day after the injury, with up to one-quarter arriving after a week. This was different from the findings of Motah et al. (Cameroon, 2014) where 64.5% of the patient came within the first 24 h after injury [9]. The high frequency of Frankel A presentation in this latest study could explain this difference. Patients with severe clinical presentation will tend to come to a specialized setting more quickly than those with minor or moderate symptoms. In addition, most of our patients went to a healthcare center where they received treatment before being referred to our hospital when symptoms persist or worsen. This was also reported by Motah et al. (Cameroon, 2014) who found 67.7% of cases were referred from other health facilities [9]. Delay to come to a specialized center may be due to the lack of knowledge and financial constraints. Most of our patients did not have health insurance, so they would prefer to go to a nearby relatively cheap health facility.

In-hospital management in our study included three arms: medical, orthopedic, and surgical management. Close to two-thirds of the patients benefited from psychotherapy and all the patients received steroids within 24 h of presentation, and analgesics. Less than half of our patients benefited from in-hospital physiotherapy. DGH comprises a physiotherapy unit with professionals available on demand [6]. Close to half of the patients benefited from surgery and the most common procedure was decompressive laminectomy with fixation (posterior approach only). This procedure is commonly used for TLSI patients in Cameroon [6]. In high-income countries like Germany, patients can benefit from a combined approach [16].

In this study, the median time between arrival and surgery was 120 h (5 days) and about two-thirds of the patients had surgery after a week post-injury with a median time between injury and surgery of about 8 days. This finding was consistent with that of Lofvenmark et al. (Botswana, 2014) where they had a median time between injury and surgery of 12 days [18]. Reinhold et al. (Germany, 2010) reported the median time between injury and surgery was 2 days [16]. This difference could be explained by a better pre-hospital organization with the availability of medicalized transportation, the updated technical platform, and expertise in high-income countries.

We had a mortality rate of 5.7%. This was contrary to the findings of Obalum et al. (Nigeria, 2009) where they had 17.5% [5]. This difference could be because their study included cervical spine trauma which has a higher mortality rate. In our study, respiratory failure caused 50% of deaths. This was consistent with the findings of Kawu et al. (Nigeria, 2011) where respiratory failure was the most common cause of death, responsible for 44% [19]. Efforts to improve respiratory function in patients with spine trauma improve mortality [20–22]. In our study about nine in ten patients had complications, with neurogenic pain being the most common secondary complication followed by depression (44%). This was consistent with the study of Kawu et al. (Nigeria, 2011) where they had complications in 94.8% of cases with neurogenic pain being the most prevalent and depression present in 38.4% [19]. We found that six out of every ten patients improved out of which more than two-thirds (76%) of them were in complete recovery. This was consistent with the findings of Motah et al. (Cameroon, 2013) where they had a 53.6% improvement rate though only 50% of these patients had complete recovery [6]. This difference in complete recovery could be explained by the fact that the majority of their patients came with Frankel A unlike in our case where the majority had Frankel E. In our study, the median hospital stay was 11 days (IQR: 5–26.5). The average hospital stay was 20 days (ranging from 1 day to 162 days). This was consistent with the findings of Reinhold et al. (Germany, 2010) where the average hospital stay was 19 days [16]. This was however contrary to the findings of Obalum et al. (Nigeria, 2009) where they had an average of 12 weeks [5]. This difference could be due to the fact that the majority of their patients were Frankel A while in our study, the majority had an incomplete injury (Frankel B – D).

We found an association between the degree of neurological deficit and the type of complications the patients had. More than two-thirds of patients who had bedsores had complete spinal cord injuries. This could be explained by the fact they were bedridden and our facility is deficient in terms of nurses trained in the management of patients with TLST. So bedsore preventive measures are usually delegated to the carers of the patient. Also, we do not have equipment specific for such patients like anti-bedsore mattresses. We focused on aspects of management that would improve neurological status. Here, we found that patients referred were two times more likely to have a stationary neurological status upon discharge (47.1% vs. 21.9%). This could be explained by the fact that more time was taken at the initial health center before referring to a specialized center hence worsening the lesion. Also, we found that patients who were insured were 2 times more likely to improve than those who paid cash (87.5% vs. 44.1%). This could be explained by the quality of care they received and the reduced time between arrival and surgery as money was readily available. In our study, patients who underwent in-hospital rehabilitation were 1.5 times more likely to improve than those who did not (79.3% vs. 54.1%). This was consistent with the findings of Reinhold et al. (Germany, 2010) where neurological status was improved by rehabilitation [16]. There was no significant association between the timing of surgery and neurological status upon discharge this is consistent with the work of Julian et al. (USA, 1999) where early surgery did not improve neurological status significantly compared to late surgery [23]. However, this was contrasting with the finding of Rosenfeld et al. (USA, 1998) which suggested early surgery improved the neurological status of patients significantly [24]. The difference could be explained by the fact that their study was only on the cervical spine while ours was on the thoracolumbar spine. Also, all our patients had surgery 48 h post-injury. We found referral to be a predictor of a stationary status upon discharge and being insured a predictor of an improved neurological status upon discharge. We had no management predictors of lengthy hospital stay. However, having surgery, the time between injury and surgery greater than one week, and in-hospital rehabilitation were all significantly associated with a lengthy hospital stay. This is consistent with the findings of Mckinley et al. (USA, 2004) which revealed that those who had surgery later had a longer hospital stay than those who had early surgery [25]. We also found a significant association between complications and length of hospital stay. Neurogenic pain, bedsore, wound infection, depression, and constipation were all associated with a lengthy hospital stay.

## Conclusion

The incidence of TLSI in the DGH is on the rise and it mostly affects the young male population. The most common mechanism is RTA. The arrival time at the DGH (a spine-specialized center) and in-hospital delay time for surgery at the DGH were high. Despite a resource constraint setting, many treatment options are available for patients.

## Data Availability

The data sets supporting the findings of this study are available and can be provided by the corresponding author upon reasonable request.

## Abbreviations

TLSI: Thoracolumbar Spine Injury
CT scan: Computed Tomography Scan
DGH: Douala General Hospital
RTA: Road Traffic Accident
RTC: Road Traffic Crash
MRI: Magnetic Resonance Imaging
